# Inhibitory Effects of Sodium Alginate on Hepatic Steatosis in Mice Induced by a Methionine- and Choline-Deficient Diet

**DOI:** 10.3390/md17020104

**Published:** 2019-02-09

**Authors:** Shoji Kawauchi, Sayo Horibe, Naoto Sasaki, Toshihito Tanahashi, Shigeto Mizuno, Tsuneo Hamaguchi, Yoshiyuki Rikitake

**Affiliations:** 1 Education and Research Center for Clinical Pharmacy, Kobe Pharmaceutical University, 4-19-1 Motoyamakitamachi, Higashinada-ku, Kobe 658-8558, Japan; tsuneo-h@kobepharma-u.ac.jp; 2 Laboratory of Medical Pharmaceutics, Kobe Pharmaceutical University, 4-19-1 Motoyamakitamachi, Higashinada-ku, Kobe 658-8558, Japan; s-horibe@kobepharma-u.ac.jp (S.H.); n-sasaki@kobepharma-u.ac.jp (N.S.); tanamasaki@gmail.com (T.T.); s-mizuno@med.kindai.ac.jp (S.M.); rikitake@kobepharma-u.ac.jp (Y.R.); 3 Division of Gastroenterology, Department of Internal Medicine, Kobe University Graduate School of Medicine, 7-5-1 Kusunoki-cho, Chuo-ku, Kobe 650-0017, Japan; 4 Endoscopy Department, Kindai University Nara Hospital, 1248-1 Otoda-cho, Ikoma 630-0293, Japan

**Keywords:** nonalcoholic steatohepatitis, sodium alginate, methionine and choline deficient, intestinal barrier function

## Abstract

Nonalcoholic steatohepatitis (NASH) progresses from nonalcoholic fatty liver disease (NAFLD); however, efficacious drugs for NASH treatment are lacking. Sodium alginate (SA), a soluble dietary fiber extracted from brown algae, could protect the small intestine from enterobacterial invasion. NASH pathogenesis has been suggested to be associated with enterobacterial invasion, so we examined the effect of SA on methionine- and choline-deficient (MCD) diet-induced steatohepatitis in mice (the most widely-used model of NASH). The mice (*n* = 31) were divided into three groups (mice fed with regular chow, MCD diet, and MCD diet premixed with 5% SA) for 4 and 8 weeks. The MCD diet increased lipid accumulation and inflammation in the liver, the NAFLD Activity Score and hepatic mRNA expression of tumor necrosis factor-α and collagen 1α1, and induced macrophage infiltration. Villus shortening, disruption of zonula occludens-1 localization and depletion of mucus production were observed in the small intestine of the MCD-group mice. SA administration improved lipid accumulation and inflammation in the liver, and impaired barrier function in the small intestine. Collectively, these results suggest that SA is useful for NASH treatment because it can prevent hepatic inflammation and fatty degeneration by maintaining intestinal barrier function.

## 1. Introduction

The number of patients with nonalcoholic fatty liver disease (NAFLD) has increased worldwide, possibly in accordance with the global increase in obesity. Nonalcoholic steatohepatitis (NASH) is a disease state that progresses from NAFLD. NASH is characterized by steatosis, inflammation, and ballooning degeneration of hepatocytes [1].

Recently, NASH has received considerable attention because it can progress to liver cirrhosis and hepatocellular carcinoma [2]. The pathogenesis of NASH is not known, but genetic factors, changes in intestinal bacterial flora, inflammation, and oxidative stress have been implicated [3,4]. The development of therapeutic agents for NASH has been studied, targeting the inhibition of inflammation and lipid accumulation in the liver. In particular, agonists of farnesoid X receptor (FXR), a nuclear receptor associated with the transport of bile acids, that reduce liver fat and fibrosis in animal models, are currently in the clinical trial stage [5]. Therefore, efficacious drugs that can be used to treat NASH are not available, but their development is awaited eagerly.

The liver and the small intestine are closely related via the enterohepatic circulation. Through the latter, damage to the small intestine is likely to influence the action of the liver. In recent years, several studies using clinical and animal models have reported that small-intestinal bacterial overgrowth and intestinal permeability are present in NASH [6,7]. Those results suggest that NASH progression is likely associated with the disruption of small intestinal barrier function (SIBF). Thus, it is thought that intestinal homeostasis could be a new therapeutic target for NASH.

Sodium alginate (SA) is a heteropolymer of d-mannuronic acid and l-guluronic acid. It is a soluble dietary fiber extracted from brown algae [8]. SA is in widespread clinical use as a hemostatic agent to treat gastrointestinal tract (GIT) bleeding caused by gastric and duodenal ulcers, erosion of the gastric mucosa, or reflux esophagitis [9]. SA has been reported to reduce mucosal damage and inflammation in animal models of inflammatory bowel disease [8].

Previously, we reported that pretreatment with SA can prevent indomethacin-induced small-intestinal injury and inflammation in mice, presumably through prevention of the infiltration of intestinal bacteria [10]. Studies have shown that small-intestinal bacterial overgrowth and increase of intestinal permeability occur in patients with NASH [6,7]. Thus, it is thought that the hepatic inflammatory response in NASH is induced through the delivery of intestinal bacteria-derived endotoxins and/or inflammatory cytokines via the portal vein. Those results suggest that SA may have a beneficial effect against NASH by reducing the SIBF impairments caused by intestinal bacteria. However, the effect of SA on NASH has yet to be reported.

Intestinal homeostasis may be a new potential therapeutic target for NASH; we therefore would like to investigate the effect of SA on NASH. Our results reveal the new effects of SA that were not previously characterized. We created a mouse model of methionine- and choline-deficient (MCD) diet-induced steatohepatitis. In this way, we investigated the effects of SA on lipid accumulation in the liver, liver injury-related parameters, expression of pro-inflammatory cytokines and macrophage infiltration in the liver, and SIBF.

## 2. Results

### 2.1. Inhibitory Effects of SA on MCD Diet-Induced Steatohepatitis and Liver Injury

**Figure 1:**
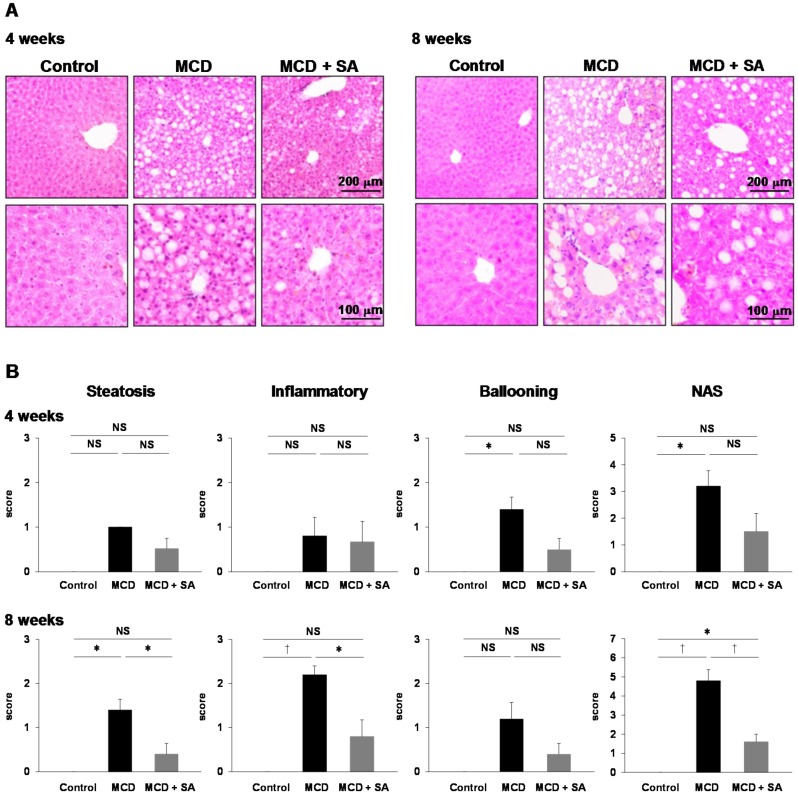
Inhibitory effects of sodium alginate (SA) on MCD diet-induced steatohepatitis in the mouse liver. (**A**) Hepatic steatosis was induced using the MCD diet for 4 and 8 weeks. In the MCD + SA group, mice were fed with the MCD diet premixed with 5% SA. Histology was undertaken using H&E staining (magnification, 100× and 200×), and representative images are shown. (**B**) The NAS was calculated as a sum of the scores of three parameters (steatosis, lobular inflammation, and hepatocellular ballooning). Data are the mean ± SEM for 5–6 mice per group. * *p* < 0.05, † *p* < 0.01, NS: not significant.

We examined the effects of SA on MCD diet-induced steatohepatitis. At 4 weeks, the Nonalcoholic Fatty Liver Disease Activity Score (NAS) with hepatocellular ballooning was increased significantly in the liver in the MCD group (*p* < 0.05, Figure 1A,B). In the MCD + SA group, the NAS tended to decrease, but there were no significant differences compared with the MCD group (Figure 1A,B). At 8 weeks, steatosis and infiltration of inflammatory cells was observed in the liver in the MCD group (Figure 1A,B).

**Figure 2:**
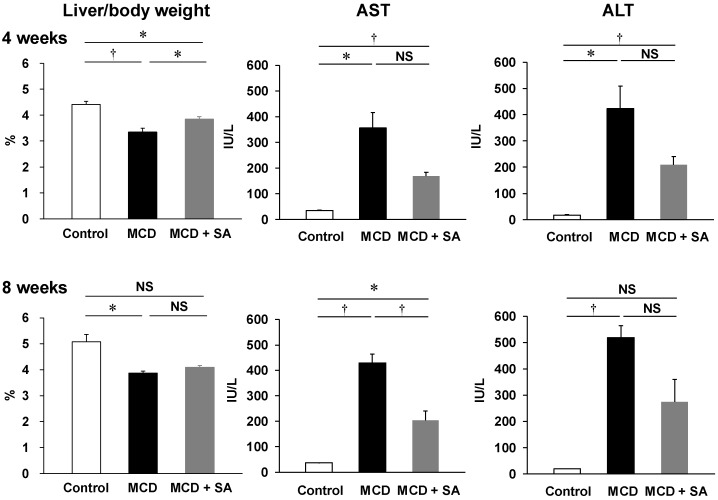
Effects of SA on liver injury-related parameters in mice with MCD diet-induced steatohepatitis. Liver weight was indicated by the liver weight:body weight ratio. Plasma levels of AST and ALT were measured according to standard biochemical methods at LSI Medience Company. Data are the mean ± SEM for 4–6 mice per group. * *p* < 0.05, † *p* < 0.01, NS: not significant.

The NAS were significantly higher in the MCD group than that in the control group (*p* < 0.01), and the NAS was significantly lower in the MCD + SA group than that in the MCD group (*p* < 0.01, Figure 1B). At 4 and 8 weeks, the liver weight in the MCD group was significantly lower than that in the control group (*p* < 0.01 and *p* < 0.05, Figure 2). At 4 weeks, the decreased liver weight was improved significantly in the MCD + SA group (*p* < 0.05, Figure 2). At 4 and 8 weeks, plasma levels of aspartate aminotransferase (AST) and alanine aminotransferase (ALT) in the MCD group were significantly higher than those in the control group (*p* < 0.05 and *p* < 0.01, Figure 2). In the MCD + SA group, plasma levels of AST and ALT tended to decrease, and the difference in plasma levels of AST at 8 weeks between the MCD group and MCD + SA group reached significance (*p* < 0.05, Figure 2). These results suggested that SA could slow or prevent the progression of MCD diet-induced steatohepatitis.

### 2.2. Effects of SA on Expression of Tumor Necrosis Factor-alpha (Tnf-α) and Collagen 1α1 mRNA and Macrophage Infiltration in the Liver of Mice with MCD Diet-Induced Steatohepatitis

**Figure 3:**
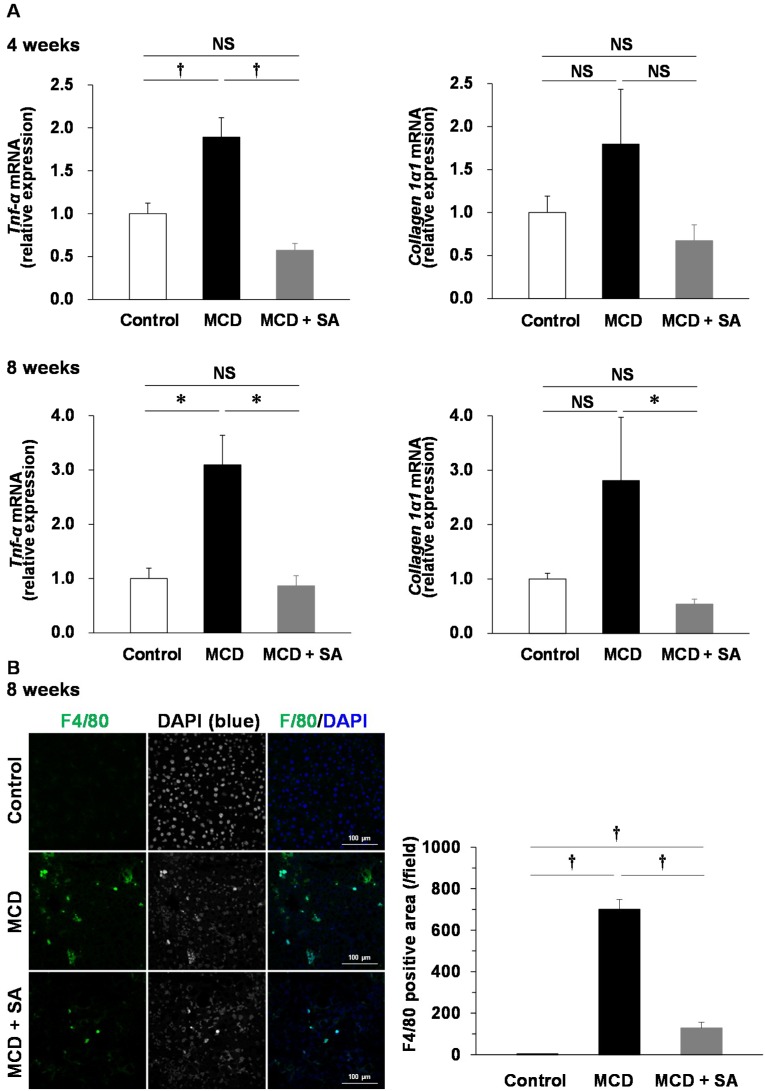
Effects of SA on expression of *Tnf-α* and *collagen 1α1* mRNA and macrophage infiltration in the liver of mice with MCD diet-induced steatohepatitis. (**A**) Expression of *Tnf-α* and *collagen 1α1* mRNA was determined using real-time PCR. Data are the mean ± SEM for 4–5 mice per group. * *p* < 0.05, † *p* < 0.01, NS: not significant. (**B**) Liver sections were stained with F4/80 (green) and DAPI (blue). Representative immunofluorescence images are shown. The F4/80-positive area per field was analyzed using ImageJ. Data are the mean ± SEM for 3 mice per group. † *p* < 0.01.

TNF-α is implicated in the pathogenesis of steatohepatitis [11]. Therefore, we measured the mRNA expression of *Tnf-α* in the liver of mice with MCD diet-induced steatohepatitis. Expression of *Tnf-α* mRNA was significantly higher at 4 and 8 weeks in the MCD group than that in the control group (*p* < 0.01 and *p* < 0.05, Figure 3A). Expression of *Tnf-α* mRNA was significantly lower in the MCD + SA group than that in the MCD group (*p* < 0.01 and *p* < 0.05, Figure 3A). The mRNA expression of *collagen 1α1*, a fibrosis marker was higher at 4 and 8 weeks in the MCD group than that in the control group. At 8 weeks, expression of *collagen 1α1* mRNA was significantly lower in the MCD + SA group than that in the MCD group (*p* < 0.05, Figure 3A). Immunofluorescence staining for F4/80-positive macrophages was significantly more intense at 8 weeks in the MCD group than that in the control group (*p* < 0.01), and fewer F4/80-positive macrophages were documented in the MCD + SA group than those in the MCD group (*p* < 0.01, Figure 3B). These results suggested that SA had preventative effects on expression of *Tnf-α* mRNA because it inhibited macrophage infiltration into the liver of mice with MCD diet-induced steatohepatitis.

### 2.3. Effects of SA on Small-Intestinal Injury in Mice with MCD Diet-Induced Steatohepatitis

NASH is associated with small-intestinal bacterial overgrowth and intestinal permeability [6,7]. Therefore, we examined SIBF by evaluating villus height, mucus production, and the architecture of tight junctions (TJs) in mice with MCD diet-induced steatohepatitis.

**Figure 4:**
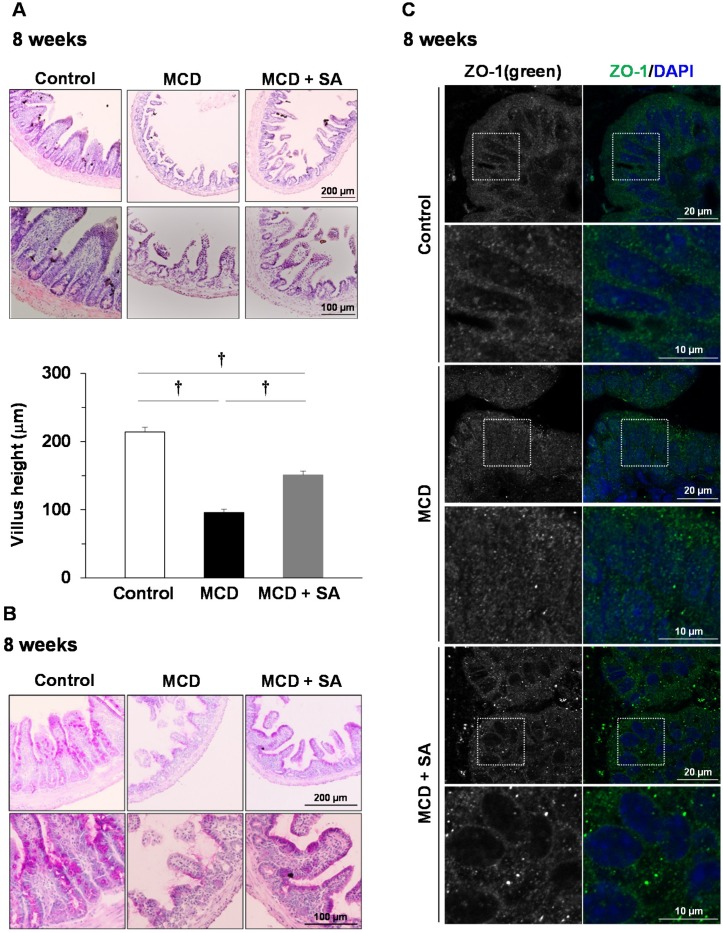
Effects of SA on small-intestinal injury in mice with MCD diet-induced steatohepatitis. (**A**) Histology was carried out using H&E staining (magnification, 100× and 200×), and representative images are shown. The height of five intact and well-oriented villi per sample was measured using ImageJ and the average value used as data. Results are the mean ± SEM for 4–6 mice per group. † *p* < 0.01. (**B**) Mucus production in the small intestine was detected using PAS staining (magnification, 100× and 200×), and representative images are shown. (**C**) Small-intestine sections were stained with ZO-1 (green) and DAPI (blue). Representative immunofluorescence images are shown. Magnified images represent high-magnification images of the boxed area.

At 8 weeks, atrophy and shortening of villi were observed in the small intestine in the MCD group (Figure 4A). In the MCD + SA group, such damage to villi was improved significantly (*p* < 0.01, Figure 4A). Depletion of periodic acid-Schiff (PAS)-positive mucus production was detected in the MCD group, and this depletion was improved in the MCD + SA group (Figure 4B). The barrier function of the small-intestinal epithelium is controlled by TJs. Zonula occludens (ZO)-1 is a well-characterized TJ protein [12]. Concentrated localization of the immunofluorescence signal for ZO-1 at villi epithelia in the small intestine was detected in the control and MCD + SA groups. In contrast, concentrated localization of a ZO-1 signal at villi epithelia was lost in the MCD group (Figure 4C). These results suggested that SA prevents SIBF impairments in mice with MCD diet-induced steatohepatitis.

## 3. Discussion

The present study showed that SA administration could slow or prevent steatohepatitis progression in mice with MCD diet-induced steatohepatitis through a mechanism involving protection against SIBF impairments.

Choline and methionine are essential components for very-low-density lipoproteins. The MCD diet, which lacks these components, can induce lipid accumulation in the liver caused by reduced lipid transport out of hepatocytes [13]. MCD diet-induced steatohepatitis is accompanied by oxidative stress, increased levels of TNF-α in the liver, and increased levels of lipids to induce a form of NASH that is very similar to that observed in humans [13]. The high fat diets (HFD) have also been widely used to induce steatohepatitis in animals [14,15]. In methionine- and choline-deficient (MCD) diets, inflammation, fibrosis and hepatocyte apoptosis develop faster and more severely than mice fed HFD. Therefore, MCD diet-induced steatohepatitis is a useful experimental model to examine the mechanism of NASH progression such as inflammation and fibrosis [16].

SA is a polysaccharide found in brown algae. It is in widespread clinical use as a hemostatic agent to treat GIT bleeding (i.e., gastric and duodenal ulcers, erosion of the gastric mucosa, and reflux esophagitis) [9]. In the present study, histology revealed that the NAS with steatosis and infiltration of inflammatory cells were significantly higher in the MCD group than those in the control group, and that this increase was reduced significantly upon SA treatment. Furthermore, plasma levels of AST in the MCD group were significantly higher than those in the control group, and this increase was reduced significantly by SA treatment.

Deficiency of TNF receptors 1 and 2 prevents the progression of lipid accumulation and fibrosis in the livers of mice with MCD diet-induced steatohepatitis [11,17]. Thus, TNF-α plays a central part in the mechanism of action of NASH. We showed that infiltration of F4/80-positive macrophages (the major sources of TNF-α) and expression of *Tnf-α* mRNA were increased in the livers of MCD-group mice, and that these features were prevented significantly in the MCD + SA group.

It has been reported that small-intestinal bacterial overgrowth, increased intestinal permeability and increased serum levels of TNF-α occur in NASH patients and experimental animals [6,7]. Ethanol production by intestinal bacteria is increased in obesity [18], which may contribute to increased small-intestinal permeability. In the NAFLD model, the probiotic *Lactobacillus rhamnosus GG* not only regulates intestinal flora but also limits the concentrations of intestinal bacteria-derived endotoxins in the portal vein, inflammation, and lipid accumulation in the liver [19]. Those studies suggest that alterations in the composition of intestinal microbiota and intestinal permeability lead to enhanced translocation of bacterial endotoxins into the portal vein [20]. Moreover, increasing sensitivity to endotoxins by the liver has been observed in obese mice because steatohepatitis and liver injury are induced by administration of low-dose lipopolysaccharide [21]. Therefore, in the hepatic inflammation and fatty degeneration observed in NASH, it is hypothesized that endotoxins that reach the liver via the portal vein activate hepatic macrophages (Kupffer cells) and induce the production of pro-inflammatory cytokines such as TNF-α.

The barrier function of the intestinal epithelium is regulated by TJs. ZO-1 is an important protein constituting TJs [12]. We showed disruption of ZO-1 localization and villi shortening in the small intestine of MCD-group mice, and that these SIBF impairments were improved upon SA treatment. Pathologic changes in villi and down-regulation of TJ expression are associated with steatohepatitis progression [22], which is in accordance with our findings.

SA undergoes very little absorption by the GIT, so its anti-ulcer action is thought to be exerted locally in ulcerative lesions [23,24]. SA-induced mucin production can inhibit enterobacterial invasion from sites of small-intestinal injury caused by non-steroidal anti-inflammatory drugs [25]. In the present study, depletion of PAS-positive mucus production was detected in the MCD group compared with the control group, and such depletion was improved upon SA treatment.

**Figure 5:**
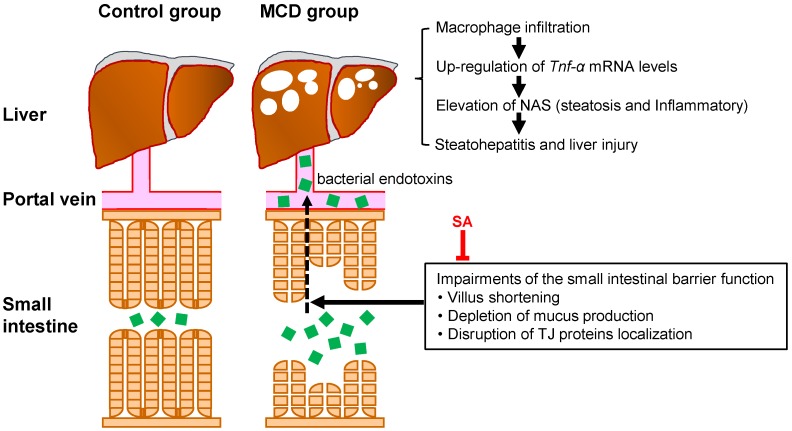
Model for the mechanisms of protective effects of SA on MCD diet-induced steatohepatitis in the mouse liver.

Collectively, our data suggest that SA prevents the hepatic inflammation and fatty degeneration observed in mice with MCD diet-induced steatohepatitis by maintaining SIBF (Figure 5). However, further studies are needed to determine the exact mechanism of the inhibitory effects of SA on steatohepatitis progression.

It is thought that the hepatic inflammatory response in NASH is induced through the delivery of intestinal bacteria-derived endotoxins and/or inflammatory cytokines via the portal vein. The MCD diet induced not only macrophage infiltration, increase in expression of *Tnf-α* mRNA, and increase in the NAS in the liver, but also villus shortening, depletion of mucus production and disruption of localization of TJ proteins in the small intestine. SA prevented hepatic inflammation and fatty degeneration in mice with MCD diet-induced steatohepatitis by maintaining intestinal barrier function.

## 4. Materials and Methods

### 4.1. Induction of Steatohepatitis Using a MCD Diet

Animal experiments were approved by the Animal Care Committee of Kobe Pharmaceutical University (Kobe, Japan).

Male C57BL/6 mice (6 weeks) were purchased from CLEA Japan (Shizuoka, Japan). Mice were maintained under an artificial 12-h dark-light cycle (lights on at 08:30) at a constant temperature of 24 ± 1 °C and 55% humidity under specific pathogen-free conditions. They were provided with CE-2 laboratory chow (CLEA Japan) and water ad libitum for 1 week.

Then, mice were divided into three groups, in which they were fed: (i) regular CE-2 chow (control group, *n* = 10), (ii) a diet deficient in methionine and choline (Oriental Yeast Company, Tokyo, Japan) (MCD group, *n* = 10) or (iii) the MCD diet premixed with 5% SA (a kind gift from Kaigen Pharma (Osaka, Japan)) (MCD + SA group, *n* = 11).

After 4 or 8 weeks, the mice were deeply anesthetized, and then the liver was excised rapidly and used for experimentation.

### 4.2. Histology

Samples of liver tissue were fixed using neutral-buffered 10% formaldehyde and then embedded in paraffin to form a tissue block. Sections (thickness, 4 µm) were prepared, stained using hematoxylin and eosin (H&E), and then evaluated microscopically for hepatic lipid accumulation. Hepatic pathology was assessed by the NAS comprising three factors: steatosis (0 to 3); lobular inflammation (0 to 3); hepatocellular ballooning (0 to 2) [1].

The samples of small-intestinal tissue were embedded in OCT compound (Sakura Finetek, Tokyo, Japan) to form a tissue block. Sections of thickness 10 µm were stained using H&E. Villus height was measured as reported previously, with modifications [26].

Five intact and well-oriented villi, from the top of the villus to the villus-crypt junction, were measured and averaged for each sample using a light microscope (BX50; Olympus Corporation, Tokyo, Japan). Mucus production in the small intestine was detected using PAS staining.

### 4.3. Real-Time Reverse-Transcription Polymerase Chain Reaction (RT-PCR)

Total RNA was extracted from the liver with TRIzol™ Reagent according to the manufacturer’s (Life Technologies, Carlsbad, CA, USA) instructions. The quantity of extracted RNA was determined using a ND-1000 spectrophotometer (NanoDrop Technologies, Wilmington, DE, USA). A total of 200 ng of RNA was subjected to a RT reaction. Purified total RNA was reverse-transcribed using a ReverTra Ace^®^ qPCR RT kit (Toyobo, Osaka, Japan) in a total volume of 20 µL. For real-time PCR amplification, 2 µL of cDNA was used as a template. PCR amplification was done using a Thermal Cycler Dice Real Time System (Takara Bio, Otsu, Japan) with Thunderbird SYBR qPCR Mix (Toyobo). PCR conditions were: 40 cycles of 15 s at 95 °C and 45 s at 60 °C. The PCR primer sequences (forward and reverse, respectively) used for detecting expression of *Tnf-α*, *Collagen 1α1*, and *Actb* were 5′-CCACCACGCTCTTCTGTCTAC-3′ and 5′-AGGGTCTGGGCCATAGAACT-3′, 5′- GAGAGAGCATGACCGATGGATT-3′ and 5′-TGTAGGCTACGCTGTTCTTGCA-3′, and 5′-AGAGGGAAATCGTGCGTGAC-3′ and 5′- CAATAGTGATGACCTGGCCGT-3′, respectively [27,28,29].

### 4.4. Analyses of Biochemical Data

Blood samples were collected from the inferior vena cava. Plasma was isolated through centrifugation at 3500× *g* for 5 min at room temperature. Levels of AST and ALT in plasma were analyzed according to standard biochemical methods at LSI Medience Company (Tokyo, Japan).

### 4.5. Immunofluorescence Microscopy

Sections of OCT compound-embedded liver and small-intestine tissue (10 µm) were incubated with 1% bovine serum albumin, 10% normal goat serum, and 0.5% Triton X-100 in phosphate-buffered saline for 60 min at room temperature. Sections of liver and small-intestine tissue were stained, respectively, with the primary antibodies anti-F4/80-Alexa Fluor® 488 conjugate (1:100 dilution; Abcam, Cambridge, UK; ab6640, RRID:AB_1140040) or anti-rabbit polyclonal ZO-1 antibody (1:100 dilution; Thermo Fisher Scientific, Waltham, MA, USA; 40-2300, AB_2533457) at room temperature overnight. Then, sections of small-intestine tissue were stained with secondary antibodies (goat anti-rabbit Alexa Fluor 488; 1:200 dilution; Thermo Fisher Scientific; A-11034, AB_2576217) for 7 h at room temperature. Sections were mounted in Prolong^®^ Diamond Antifade Reagent with 4′,6-diamidino-2-phenylindole (Thermo Fisher Scientific) and the slide protected with cover glass. Image analyses of immunofluorescent staining were done on a confocal laser scanning microscope (LSM700; Carl Zeiss, Oberkochen, Germany). Images captured on the confocal laser scanning microscope were analyzed using ZEN acquisition software (ZEN 2.3 SP1 (black); Carl Zeiss, Oberkochen, Germany).

### 4.6. Statistical Analyses

Data represent the mean ± standard error of independent determinations for each experiment. Significance was analyzed using one-way analysis of variance, followed by the Tukey-Kramer test or Dunnett’s T3 test using SPSS v20.0 (IBM, Armonk, NY, USA) when the data were normally distributed. If the data were not distributed normally, then the Kruskal-Wallis test was used for analysis. *p* < 0.05 was considered significant.

## 5. Conclusions

SA could slow or prevent lipid accumulation and inflammation in the liver of mice with MCD diet-induced steatohepatitis. Our results suggest that intestinal homeostasis could be a new therapeutic target for NASH, in addition to inhibition of liver inflammation and lipid accumulation. In addition, it is important in terms of drug repositioning that new uses for existing drugs are found. Therefore, we propose SA as a potential therapeutic agent for NASH, for which an effective therapeutic agent is lacking.

## References

[1] Kleiner D.E., Brunt E.M., Van Natta M., Behling C., Contos M.J., Cummings O.W., Ferrell L.D., Liu Y.C., Torbenson M.S., Unalp-Arida A. (2005). Design and validation of a histological scoring system for nonalcoholic fatty liver disease. Hepatology.

[2] Ascha M.S., Hanouneh I.A., Lopez R., Tamimi T.A., Feldstein A.F., Zein N.N. (2010). The incidence and risk factors of hepatocellular carcinoma in patients with nonalcoholic steatohepatitis. Hepatology.

[3] Tilg H., Moschen A.R. (2010). Evolution of inflammation in nonalcoholic fatty liver disease: The multiple parallel hits hypothesis. Hepatology.

[4] Trauner M., Arrese M., Wagner M. (2010). Fatty liver and lipotoxicity. Biochim. Biophys. Acta.

[5] Neuschwander-Tetri B.A., Loomba R., Sanyal A.J., Lavine J.E., Van Natta M.L., Abdelmalek M.F., Chalasani N., Dasarathy S., Diehl A.M., Hameed B. (2015). Farnesoid X nuclear receptor ligand obeticholic acid for non-cirrhotic, non-alcoholic steatohepatitis (FLINT): A multicentre, randomised, placebo-controlled trial. Lancet.

[6] Wigg A.J., Roberts-Thomson I.C., Dymock R.B., McCarthy P.J., Grose R.H., Cummins A.G. (2001). The role of small intestinal bacterial overgrowth, intestinal permeability, endotoxaemia, and tumour necrosis factor alpha in the pathogenesis of non-alcoholic steatohepatitis. Gut.

[7] Luther J., Garber J.J., Khalili H., Dave M., Bale S.S., Jindal R., Motola D.L., Luther S., Bohr S., Jeoung S.W. (2015). Hepatic injury in nonalcoholic steatohepatitis contributes to altered intestinal permeability. Cell Mol. Gastroenterol. Hepatol..

[8] Yamamoto A., Itoh T., Nasu R., Nishida R. (2013). Effect of sodium alginate on dextran sulfate sodium- and 2,4,6-trinitrobenzene sulfonic acid-induced experimental colitis in mice. Pharmacology.

[9] Poynard T., Vernisse B., Agostini H. (1998). Randomized, multicentre comparison of sodium alginate and cisapride in the symptomatic treatment of uncomplicated gastro-oesophageal reflux. Aliment Pharmacol. Ther..

[10] Horibe S., Tanahashi T., Kawauchi S., Mizuno S., Rikitake Y. (2016). Preventative effects of sodium alginate on indomethacin-induced small-intestinal injury in mice. Int. J. Med. Sci..

[11] Li Z., Yang S., Lin H., Huang J., Watkins P.A., Moser A.B., Desimone C., Song X.Y., Diehl A.M. (2003). Probiotics and antibodies to TNF inhibit inflammatory activity and improve nonalcoholic fatty liver disease. Hepatology.

[12] González-Mariscal L., Betanzos A., Avila-Flores A. (2000). MAGUK proteins: Structure and role in the tight junction. Semin. Cell Dev. Biol..

[13] Marcolin E., Forgiarini L.F., Tieppo J., Dias A.S., Freitas L.A., Marroni N.P. (2011). Methionine- and choline-deficient diet induces hepatic changes characteristic of non-alcoholic steatohepatitis. Arq. Gastroenterol..

[14] Park E.Y., Choi H., Yoon J.Y., Lee I.Y., Seo Y., Moon H.S., Hwang J.H., Jun H.S. (2015). Polyphenol-rich fraction of ecklonia cava improves nonalcoholic fatty liver disease in high fat diet-fed mice. Mar. Drugs.

[15] Gabbia D., Dall’Acqua S., Di Gangi I.M., Bogialli S., Caputi V., Albertoni L., Marsilio I., Paccagnella N., Carrara M., Giron M.C. (2017). The phytocomplex from fucus vesiculosus and ascophyllum nodosum controls postprandial plasma glucose levels: An in vitro and in vivo study in a mouse model of NASH. Mar. Drugs.

[16] Machado M.V., Michelotti G.A., Xie G., Almeida Pereira T., Boursier J., Bohnic B., Guy C.D., Diehl A.M. (2015). Mouse models of diet-induced nonalcoholic steatohepatitis reproduce the heterogeneity of the human disease. PLoS ONE.

[17] Tomita K., Tamiya G., Ando S., Ohsumi K., Chiyo T., Mizutani A., Kitamura N., Toda K., Kaneko T., Horie Y. (2006). Tumour necrosis factor alpha signalling through activation of Kupffer cells plays an essential role in liver fibrosis of non-alcoholic steatohepatitis in mice. Gut.

[18] Cope K., Risby T., Diehl A.M. (2000). Increased gastrointestinal ethanol production in obese mice: Implications for fatty liver disease pathogenesis. Gastroenterology.

[19] Ritze Y., Bárdos G., Claus A., Ehrmann V., Bergheim I., Schwiertz A., Bischoff S.C. (2014). *Lactobacillus rhamnosus* GG protects against non-alcoholic fatty liver disease in mice. PLoS ONE.

[20] Endo H., Niioka M., Kobayashi N., Tanaka M., Watanabe T. (2013). Butyrate-producing probiotics reduce nonalcoholic fatty liver disease progression in rats: New insight into the probiotics for the gut-liver axis. PLoS ONE.

[21] Yang S.Q., Lin H.Z., Lane M.D., Clemens M., Diehl A.M. (1997). Obesity increases sensitivity to endotoxin liver injury: Implications for the pathogenesis of steatohepatitis. Proc. Natl. Acad. Sci. USA.

[22] Peng J.H., Cui T., Huang F., Chen L., Zhao Y., Xu L., Xu L.L., Feng Q., Hu Y.Y. (2013). Puerarin ameliorates experimental alcoholic liver injury by inhibition of endotoxin gut leakage, Kupffer cell activation, and endotoxin receptors expression. J. Pharmacol. Exp. Ther..

[23] Humphreys E.R., Triffitt J.T. (1968). Absorption by the rat of alginate labelled with carbon-14. Nature.

[24] Daigo K., Wada Y., Yamada C., Yamaji M., Okuda S., Okada M., Miyazato T. (1981). Pharmacological studies of sodium alginate. I. Protective effect of sodium alginate on mucous membranes of upper-gastrointestinal tract. Yakugaku Zasshi.

[25] Yamamoto A., Itoh T., Nasu R., Nishida R. (2014). Sodium alginate ameliorates indomethacin-induced gastrointestinal mucosal injury via inhibiting translocation in rats. World J. Gastroenterol..

[26] Kato S., Hamouda N., Kano Y., Oikawa Y., Tanaka Y., Matsumoto K., Amagase K., Shimakawa M. (2017). Probiotic Bifidobacterium bifidum G9-1 attenuates 5-fluorouracil-induced intestinal mucositis in mice. via suppression of dysbiosis-related secondary inflammatory responses. Clin. Exp. Pharmacol. Physiol..

[27] Baluk P., Yao L.C., Feng J., Romano T., Jung S.S., Schreiter J.L., Yan L., Shealy D.J., McDonald D.M. (2009). TNF-alpha drives remodeling of blood vessels and lymphatics in sustained airway inflammation in mice. J. Clin. Investig..

[28] Overbergh L., Valckx D., Waer M., Mathieu C. (1999). Quantification of murine cytokine mRNAs using real time quantitative reverse transcriptase PCR. Cytokine.

[29] Kong B., Luyendyk J.P., Tawfik O., Guo G.L. (2009). Farnesoid X receptor deficiency induces nonalcoholic steatohepatitis in low-density lipoprotein receptor-knockout mice fed a high-fat diet. J. Pharmacol. Exp. Ther..

